# The British Journals: Midwifery

**Published:** 1839-10

**Authors:** 


### MIDWIFERY.

*Case of Restoration of Animation in a Stillborn Child.* By H. Terry, Esq., Surgeon to the Northampton Infirmary.

This is a very interesting case, and adds to the credit already acquired by Mr. Terry for his long and successful prosecution of this important subject. We must find room for a brief extract. The infant was stillborn; and from fifteen to twenty minutes had elapsed ere Mr. Terry saw it. "The body of the child did not present the slighest appearance of life, and I thought the prospect of restoration was feeble in the extreme. It was right, however, to attempt it. Having set the funis at liberty, I hastily wiped the child's mouth, and at once began breathing into its lungs. I have various tubes for this purpose, but I never use any one of them: I find, or at least fancy, that nothing is so efficacious as my own mouth, and for this purpose I usually have with me a bit of leno or coarse muslin to place over the child's mouth to render the work a little less disagreeable. I continued breathing, with the child in this situation, for a minute or two, and then, observing the funis quite cold and collapsed, I hastily separated it, got the child into hot water, and gave myself a more convenient and agreeable situation for continuing the process. I am accustomed to keep the baby up to its chin in hot water, if the vessel will admit of it; if not, I cover the upper part of the body with flannels kept continually hot and and wet with the water. I support the head with my left hand, and compress the chest after each inflation of the lungs with my right hand, employing an attendant to hold the child's nose, if I find it necessary. In about ten minutes a very faint sob or slight attempt at inspiration took place, and shortly after this I found a feeble pulsation of the heart. The process was now persevered in with the pleasing assurance of success, and at the end of about twenty or five and twenty minutes there was sufficient respiration to admit of my service being discontinued." *Medical Gazette. July* 27, 1839.

---

*Researches in Operative Midwifery.* No. 2. *Version or Turning.* By FLEETWOOD CHURCHILL, M.D., Physician to the Western Lying-in Hospital and Dispensary, Dublin.

THIS, like the preceding productions of the same author, is a most elaborate production, giving a complete history, statistical and practical, of the important operation of "Turning." The following is the actual and relative frequency of the operation in the practice of the three principal European nations. "The records of English practice yield 36,569 cases, and 135 cases of version, or 1 in $267\frac{1}{2}$. French practice 50,024 cases, and 514 cases of version, or 1 in $97\frac{1}{4}$. And German practice 21,415 cases, and 347 cases of version, or 1 in $61\frac{1}{4}$. The whole number of cases is 107, 978, and of version 996, or 1 in $111\frac{752}{966}$." We recommend this paper to the attention of all accoucheurs. *Dublin Journ. of Med. Science. July,* 1839.

